# Real Time Assays for Quantifying Cytotoxicity with Single Cell Resolution

**DOI:** 10.1371/journal.pone.0066739

**Published:** 2013-06-24

**Authors:** Sonny C. Hsiao, Hong Liu, Taylor A. Holstlaw, Cheng Liu, Catherine Y. Francis, Matthew B. Francis

**Affiliations:** 1 Adheren, Inc., Berkeley, California, United States of America; 2 Eureka Therapeutics, Emeryville, California, United States of America; 3 Department of Chemistry, University of California, Berkeley, California, United States of America; 4 Materials Sciences Division, Lawrence Berkeley National Laboratories, Berkeley, California, United States of America; University of Pécs Medical School, Hungary

## Abstract

A new live cell-based assay platform has been developed for the determination of complement dependent cytotoxicity (CDC), antibody dependent cellular cytotoxicity (ADCC), and overall cytotoxicity in human whole blood. In these assays, the targeted tumor cell populations are first labeled with fluorescent Cell Tracker dyes and immobilized using a DNA-based adhesion technique. This allows the facile generation of live cell arrays that are arranged arbitrarily or in ordered rectilinear patterns. Following the addition of antibodies in combination with serum, PBMCs, or whole blood, cell death within the targeted population can be assessed by the addition of propidium iodide (PI) as a viability probe. The array is then analyzed with an automated microscopic imager. The extent of cytotoxicity can be quantified accurately by comparing the number of surviving target cells to the number of dead cells labeled with both Cell Tracker and PI. Excellent batch-to-batch reproducibility has been achieved using this method. In addition to allowing cytotoxicity analysis to be conducted in real time on a single cell basis, this new assay overcomes the need for hazardous radiochemicals. Fluorescently-labeled antibodies can be used to identify individual cells that bear the targeted receptors, but yet resist the CDC and ADCC mechanisms. This new approach also allows the use of whole blood in cytotoxicity assays, providing an assessment of antibody efficacy in a highly relevant biological mixture. Given the rapid development of new antibody-based therapeutic agents, this convenient assay platform is well-poised to streamline the drug discovery process significantly.

## Introduction

Antibodies are able to recognize and destroy targeted cells, such as those corresponding to tumors or viral infections, through complement dependent cytotoxicity (CDC) and antibody dependent cellular cytotoxicity (ADCC) [1], [2]. These pathways are believed to be involved in the mechanism of action for many antibody-based therapeutics, and thus it is imperative to be able to assess the ability of an immunoglobulin drug candidate to elicit these responses. Cell lysis by the CDC or ADCC process is typically measured for a bulk population by monitoring the release of chromium-51 (^51^Cr) [3] that had been previously taken up by the cells, or by the release of lactic acid dehydrogenase (LDH) [4], [5]. The measurement of cell viability has also been successfully measured for a CDC experiment using a soluble MTT reporter [6], [7]. Although widely used, however, all of these methods have their shortcomings. ^51^Cr is radioactive, expensive, and adds disposal difficulties, which has led to the popularity of the LDH release method. However, this method can lead to large sample errors in ADCC assays since both the target and effector cells contain LDH [8], [9]. All of the available methods provide an average toxicity value for a whole population of cells, providing no information about individual cell behavior. Finally, these techniques require the evaluation of relatively large cell populations to obtain usable reproducibility. This can be especially problematic in cases where targeted cells are in short supply, such as the use of blood samples from specific leukemia patients.

To address these limitations while providing increased amounts of diagnostic information for a particular cell-treatment combination, we report herein a new cytotoxicity assay that can be used to evaluate the response of individual cells to antibodies and other drug candidates. The technique uses fluorescence microscopy and automated image processing to determine the number of both living and dead cells with a high degree of precision, and only requires inexpensive and readily available dyes. The method can be used in real time to provide temporal information about cytotoxicity, and it can be used to identify cells that bear the targeted receptor, and yet resist the CDC and ADCC mechanisms. It can also clearly distinguish between targeted and effector cells, providing accurate cytotoxicity data using the complex samples of peripheral blood mononuclear cells (PBMCs) and even whole blood. In this work, this analysis method is demonstrated using leukemia and lymphoma cells and a known therapeutic antibody. However, the generality of the method should allow its extension to the evaluation of many different tumor cell types and drug candidates.

## Results and Discussion

### Creating Live Cell Arrays through DNA Adhesion

**Figure 1:**
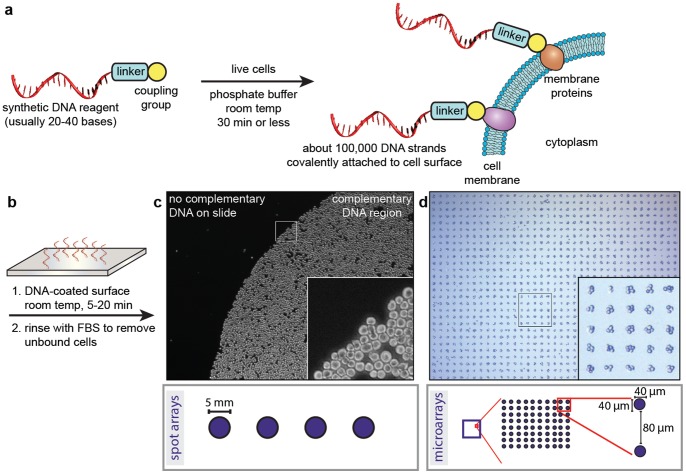
Programing cellular adhesion using DNA hybridization. (a) The exposure of live cells to an activated DNA reagent results in the rapid attachment of single strands to proteins in the cytoplasmic membrane. (b) Cell-substrate adhesion occurs within 5–20 min upon contact with a surface that displays the complementary DNA sequence. (c) The result of this process is shown for a human T-cell cancer line (Jurkat) bound to a 5 mm DNA spot printed on a glass slide. Unbound cells were readily washed from the slide. (d) When combined with DNA patterns generated using photolithography, ordered rectilinear arrays of cell clusters can also be produced. Both patterns are available in glass-bottomed chamber slides or 96-well plates.

A key aspect of this technique is the attachment of living cells to analysis surfaces through the use of DNA-based adhesion [10]–[14]. In this approach, synthetic DNA strands bearing NHS esters are covalently conjugated to proteins on the surfaces of the target cells, as outlined schematically **Fig. 1a**. Previous studies have indicated that the modification procedure results in the addition of ∼100,000 DNA strands, with no notable effects on cell morphology or undesired pathway activation. Upon exposure to glass surfaces bearing the sequence complements, the cells adhere through DNA hybridization, **Fig. 1b**. This provides a robust linkage that can be maintained for days if needed, while maintaining excellent cell viability. In previous studies using AFM, we have measured the strength of adhesion to be ∼800 pN [14]. The use of this strategy is particularly important for cells of the immune system, as they are naturally non-adherent and thus cannot be bound using integrin-targeting strategies. Through the use of commercially available pre-printed platforms, large areas with high cell densities can be prepared (**Fig. 1c**), as well as ordered microarrays comprising small cell clusters (**Fig. 1d**). Both 96-well plate and 8-well chamber slide formats are currently available, and can be used interchangeably in the experiments described below. Following generation of the arrays, a brief rinse with PBS removes any non-bound cells. In the case of the cell microarrays, the use of this procedure provides 5000±550 (11% standard deviation for N = 5 samples) cells per well.

For experiments involving ADCC or whole blood toxicity assays (see below), the targeted cells are also labeled with fluorescent Cell Tracker dyes during the DNA modification step. This labeling is not necessary for CDC assays, as no additional cells are added in these procedures.

### CDC Assays using Live Cell Arrays

**Figure 2:**
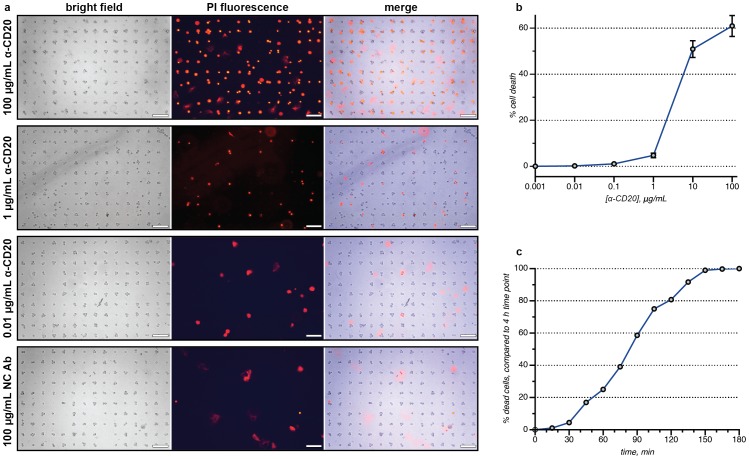
Cell-array CDC assays. (a) Microarrays of treated Jeko-1 cells were stained using a solution of propidium iodide (PI) after 4 h. The dead cells were imaged using a fluorescence microscope and counted using Image-J software, or quantified using an ImageXpress Micro automatic imager. The merged bright field and PI images facilitated the unambiguous identification of the dead cells (see close-up image in Figure 3a). The images labeled “NC Ab” correspond to a control antibody that did not bind the targeted cells. The scale bar in each image represents 100 µm. (b) A dose-dependent response was observed for an anti-CD20 antibody (α-CD20). The error bars represent the standard deviation of three replicate experiments. (c) Using time-lapsed imaging, the number of dead cells can be determined as a function of time. In this representation, 100% represents the total number of dead cells observed at 4 h, which corresponds to 60% of the total cell population (as indicated on the plot in b). These data correspond to the video that is available as supporting information.

**Figure 3:**
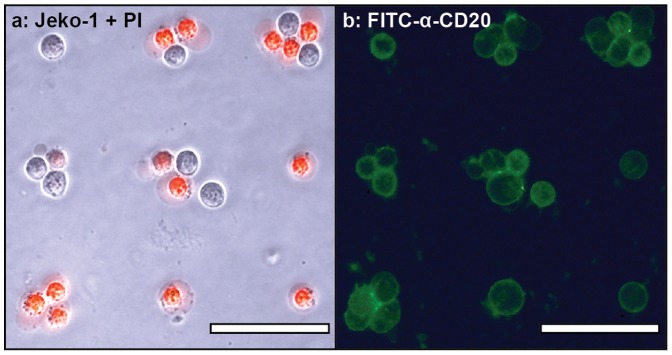
Images from a CDC assay using FITC-labeled α-CD20 at 10 µg/mL. Arrayed target cells (Jeko-1) were incubated with the antibody in human serum for 4 h, and then stained with PI. (a) A merge of the bright field and red-fluorescence (PI) images clearly indicated the dead cells. (b) The green fluorescence channel indicated that all of the target cells were bound by α-CD20. Comparison of the two images indicated which cells resisted the CDC mechanism. The scale bar in each image represents 50 µm.

To explore the use of these DNA-captured live cell populations in the context of screening therapeutic antibody candidates, different concentrations of a humanized anti-CD20 antibody (produced in CHO cells, and referred to as α-CD20 herein) [15]–[17] were applied to the wells of chamber slides containing arrays of Jeko-1 lymphoma cells. Human serum (Quidel Corporation, San Diego, CA) was then added to each well of the assay. After 4 h, the extent of CDC was determined by exposing the cells to a solution of propidium iodide (PI). By comparing bright field and fluorescence images, the number of living and dead cells could be quantitated easily, **Fig. 2a** (also see close-up image in **Fig. 3a**). Some insoluble PI stain was observed as large diffuse aggregates, but these were easily distinguished from the cells. Triplicate experiments indicated excellent plate-to-plate reproducibility, with 5% variability or less for all concentrations investigated, **Fig. 2b**. A dose-dependent response was observed, with peak toxicity seen at the highest α-CD20 concentration (100 µg/mL). Low levels of cell death, ranging between 0% to 3%, were observed when Jeko-1 cells were incubated with a negative control antibody (NC Ab), which had no affinity towards Jeko-1 cells. This result also verified that the DNA-based adhesion method itself did not lead to losses in cell viability. The overall results of this assay compared well to the previously published EC_50_ values for other anti-CD20 antibodies (such as Rituxan), which range from 5–70 µg/mL [18]–[21].

### Time-lapsed Images of the CDC Process

Because this assay has the ability to track specific cells for the full duration of the experiment, it allows one of the first real-time explorations of the CDC process. To do this, an array of DNA-immobilized Jeko-1 cells was exposed to α-CD20, serum, and PI throughout the incubation process. Time-lapsed images were acquired using an ImageXpress Micro (IXM) Automatic Imager (Molecular Devices) and merged into a movie (see **Video S1**). Cell death was observed as early as 10 min using the dose of α-CD20 (10 µg/mL), and was found to plateau after 2 h, **Fig. 2c**. The ability to acquire temporal information about the CDC process is highly useful for the evaluation of new antibody candidates, as one experiment can provide comparative information at many different time points. This allows for much greater efficiency in these assays by minimizing the number of duplicate experiments that must be run for each antibody candidate. In contrast, both the ^51^Cr- and the LDH-release assays are most suited for providing data at single endpoints.

### Identification of CDC Resistant Target Cells using a Fluorescent Antibody

The ability to conduct cytotoxicity assays on an individual cell basis provides new opportunities for the identification of drug-resistant cells [22]–[25]. Live cells can be removed from the DNA-based arrays very quickly through the addition of DNase, or somewhat more slowly through the addition of competing complementary DNA strands. Either technique provides live cells that could be used for sequencing, transcriptional profiling, or further culture.

This method is particularly informative when fluorescent antibodies are used, as cells that lack the targeted receptor can be distinguished from those that resist the CDC pathway through active mechanisms. As an example, FITC-labeled α-CD20 [26] was used to identify the receptor-positive target cells. After incubation with serum, followed by propidium iodide staining, comparison of the PI (red, **Fig. 3a**) and FITC-α-CD20 (green, **Fig. 3b**) channels revealed a number of cells that were recognized by the antibody, but nonetheless remained alive during the 4 h incubation period. This variation demonstrates the ability of this assay platform to identify individual response behaviors even within a single population of cultured cells. It is widely appreciated that much greater cell-to-cell variation is present in primary tumor tissue, and thus this method should be invaluable to groups investigating the molecular basis of drug resistance.

### Testing Antibody Specificity using Live Cell Arrays

**Figure 4:**
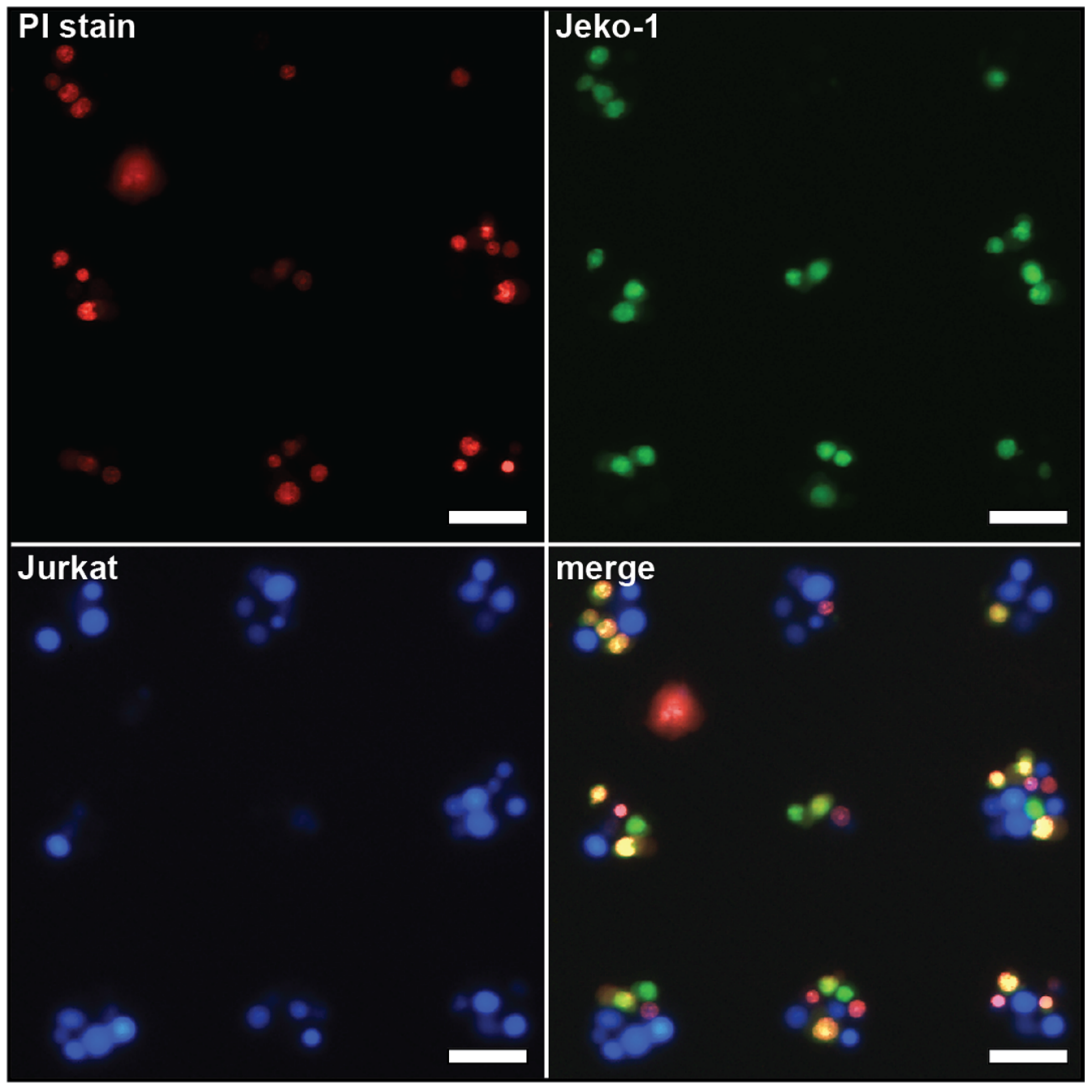
Heterotypic cellular microarrays for the determination of CDC specificity. Cell Tracker-stained Jurkat (blue channel, bottom left) and Jeko-1 (green channel, top right) cells were co-immobilized on cell microarrays. Following a CDC assay using 10 µg/mL α-CD20, the dead cells were stained red with PI (upper left). The Jeko-1 fluorescence overlapped significantly with the PI stain (yellow cells, bottom right), showing the degree of specificity of α-CD20 for CD20+ lymphoma cells. Overall, 76% of the Jeko-1 cells and 4% of the Jurkat cells were killed. The scale bar in each image represents 50 µm.

An important consideration for the development of any therapeutic antibody is the level of cytotoxicity specificity it can achieve for its cellular target. This can easily be determined using the same assay platform with fluorescence imaging at multiple wavelengths. For example, a population of Jeko-1 (a CD20-postive cell line) cells was labeled with Cell Tracker green and combined with a population of Jurkat T-leukemia cells (a CD20-negative cell line) that was labeled with Cell Tracker blue. The mixture of cells was then bound to an array of 40 µm DNA capture spots, **Fig. 4**. The CDC protocol described above was used, and the dead cells were then stained with PI. There was a clear overlap between the green (targeted) cells and the red PI stain (represented by yellow in the merged image). In contrast, the blue Jurkat cells showed little overlap with the PI stain, indicating the high degree of specificity of the α-CD20 antibody for the CD20 receptor that only the Jeko-1 cells possess. If desired, similar experimental configurations could be designed to verify the specificity of antibody binding by first labeling the immunoglobulin candidates with fluorescent dyes, as described above.

### ADCC Assays using Live Cell Arrays

**Figure 5:**
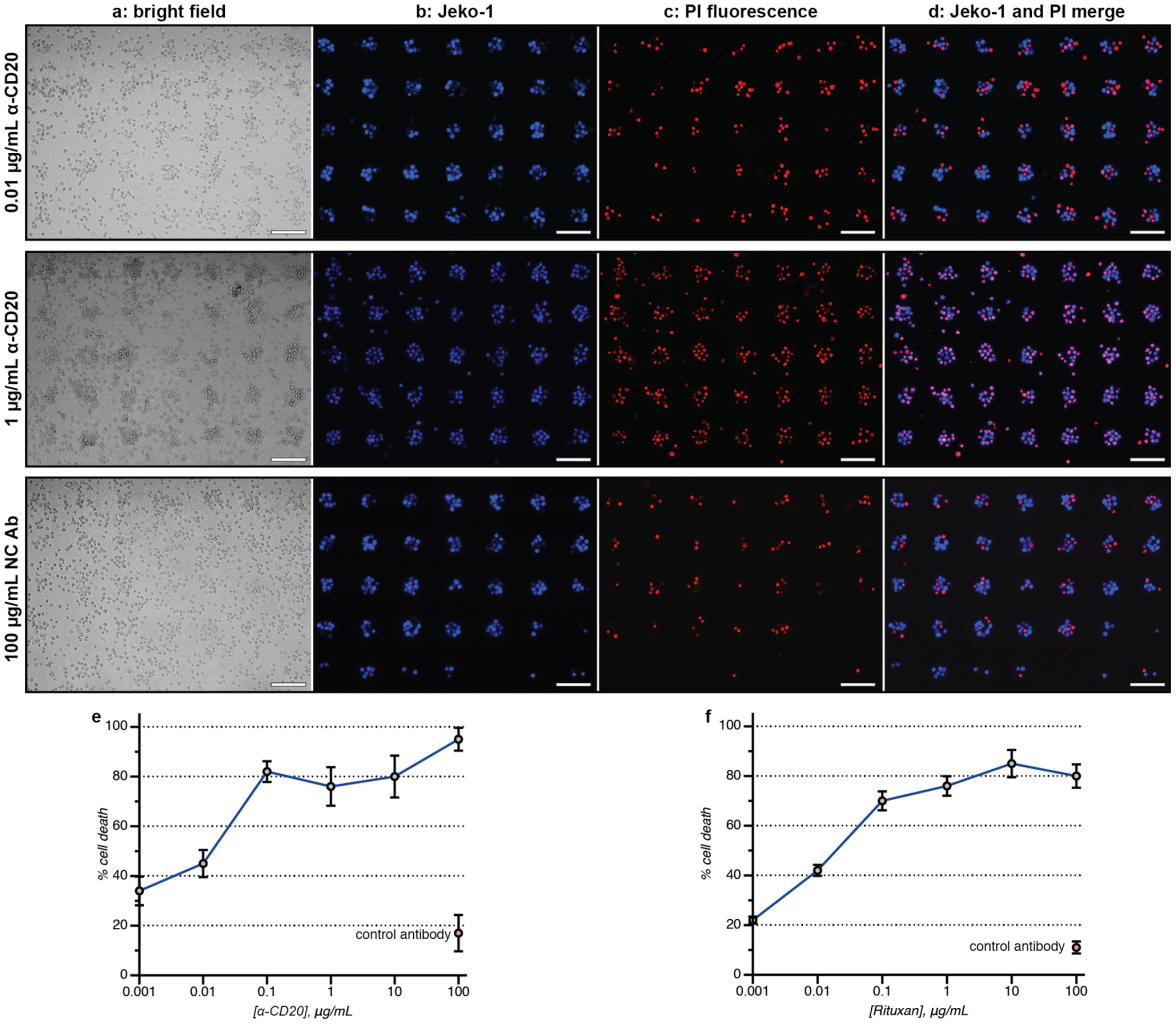
Cell-array ADCC assays. The scale bar in each image represents 100 µm. (a) The bright field images show a mixture of indistinguishable targeted cells and effector cells (PBMCs) after 16 h. (b) The Jeko-1 target cells were prestained with a Cell Tracker dye, allowing their facile detection using fluorescence microscopy. (c) The dead cells were stained red using PI and counted using Image-J software or manually. (d) A merged image allows the ratio of living (blue) to dead (purple) targeted cells to be determined. Red cells with no overlapping blue stain indicated dead effector cells, which were not counted. Cellular microarray ADCC results are shown for the dose-dependent killing of (e) Jeko-1 cells and (f) primary B-chronic lymphobatic leukemia cells with α-CD20 after 16 h. The error bars indicate the standard deviation of three replicate experiments.

A second and highly important mechanism of cytotoxicity involves the recognition of antibody Fc regions by natural killer cells. This antibody-dependent cellular cytotoxicity process is particularly difficult to carry out on a single cell basis because it is difficult to distinguish the targeted cells from effector cells supplied as mixtures of peripheral blood mononuclear cells (PBMCs). By binding only the targeted cells to the analysis surface, in addition to labeling them with cytoplasmic dyes, the DNA-based cell array method can lead to more accurate determinations of this process than were previously possible. Triplicate samples of fluorescently labeled Jeko-1 cells (blue) were arrayed in chamber slides and then exposed to the α-CD20 antibody in varying concentrations for 30 min. Suspensions of PBMCs (AllCells, Inc., 25∶1 E/T ratio) were then added directly to the samples and incubated at 37°C for 16 h. Following the addition of the PI stain, fluorescence imaging again revealed the ratio of dead to living cells, **Fig. 5a–d**. Quantitation of the resulting samples again revealed a dose-dependent response (**Fig. 5e**), with excellent plate-to-plate reproducibility (<7% variation). Nearly 100% cytotoxicity was observed at the highest α-CD20 concentration (100 µg/mL). Low levels of killing, ranging between 5% and 10%, were detected after Jeko-1 cells were incubated with a negative control antibody (NC Ab) acquired from Eureka Therapeutics, Inc. The observed ADCC EC_50_ value for α-CD20 compared well to the previously published values of 0.02–0.5 µg/mL for Rituxan [18]–[21]. These results highlight the value of being able to identify both the living and the dead target cells in a complex mixture to obtain accurate toxicity statistics. This is difficult to do using other methods. It is also possible to conduct the same assay for resistant cells in the context of an ADCC experiment when fluorescent antibodies are used, as described above for CDC assays. Similar results were obtained when primary B-chronic lymphoblastic leukemia cells were used instead of Jeko-1 cells, **Fig. 5f**.

### Cytotoxicity Assays Conducted in Whole Blood

While the ability to conduct CDC and ADCC assays independently is important for determining the mechanism of cytotoxicity, it would also be highly advantageous to evaluate the efficacy of a therapeutic antibody candidate using whole blood samples. This would effectively combine complement and cellular toxicity activity, and would provide the most relevant information regarding the specific response that a given patient would have. When combined with the ability of the cellular toxicity array method to provide accurate data using very small cell samples, this could lead to the personalized screening of antibody-based therapies against the cells obtained from a single patient. Currently the only feasible way to evaluate cytotoxicity in whole blood is the use of flow cytometry [27], which suffers from low sample throughput and requires significantly larger numbers of cells.

**Figure 6:**
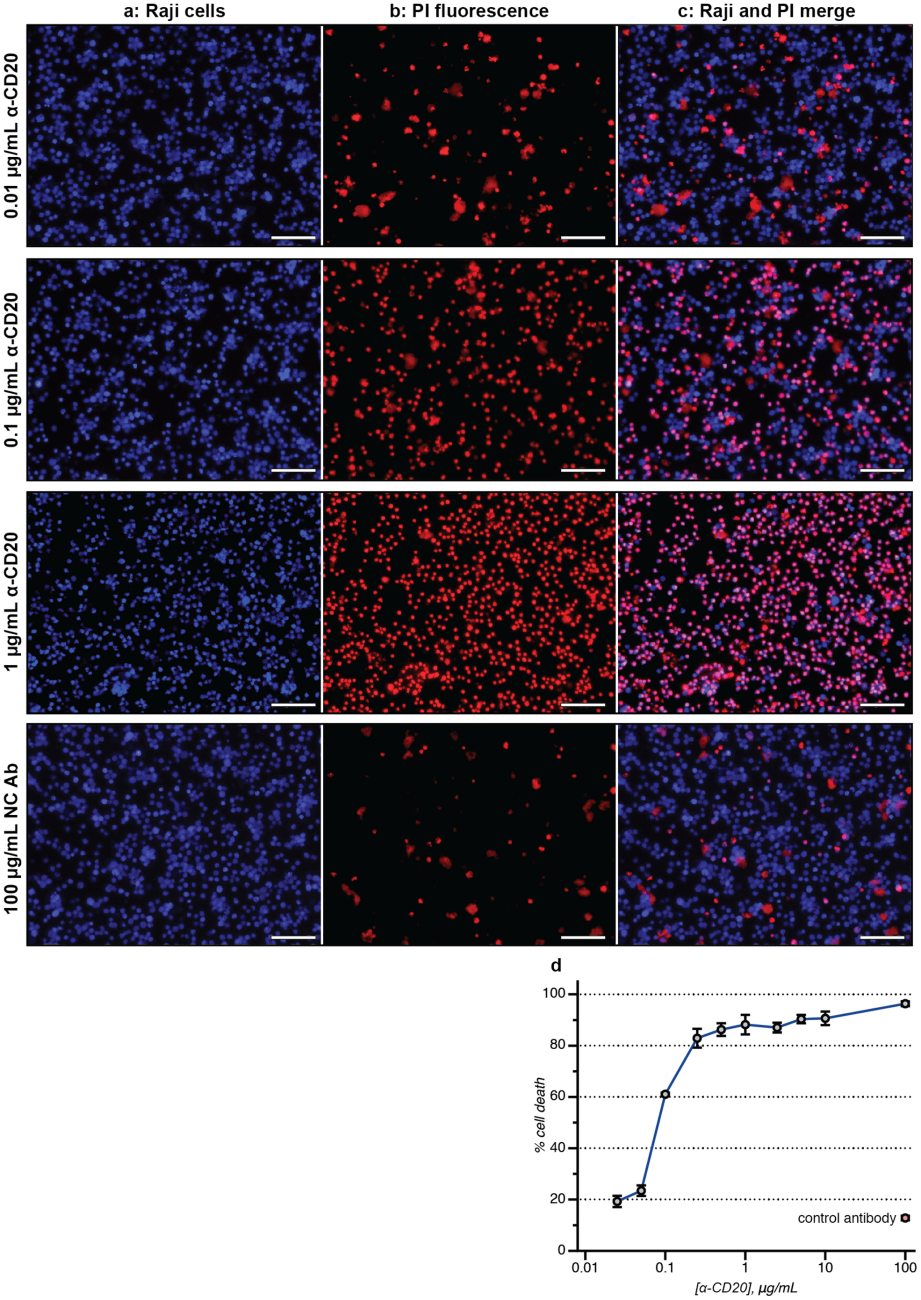
Antibody-based cytotoxicity measured in whole blood. The scale bar in each image represents 100 µm. (a) Raji lymphoma cells were labeled with a blue Cell Tracker dye and then immobilized. Varying concentrations of α-CD20 were added, followed by freshly drawn human blood. The targeted cells could be easily identified using fluorescence microscopy, even in the presence of a large excess of blood cells. (b) After 16 h, the amount of cell death could be quantified using a PI stain (red). (c) A merged image allowed the ratio of living (blue) to dead (purple) targeted cells to be determined. (d) The level of whole blood cytotoxicity is shown for α-CD20, in addition to a control antibody (red point). The error bars indicate the standard deviation of two replicate experiments.

To demonstrate this process, we first prepared a series of wells containing fluorescently labeled target cells. Large DNA capture spots were used in these assays, which removed one of the washing steps from the procedure because all of the DNA-labeled cells were bound by the plate surface. Triplicate samples (10,000 cells each) were prepared in a 96-well plate, along with 500 nM PI stain. α-CD20 was then added in varying concentrations. To each well were added 180 µL of freshly drawn human blood (AllCells, Inc.). After 16 h of incubation at 37°C, the samples were visualized directly. Because the target cells are all immobilized in the same focal plane, their fluorescence could be measured directly even without removing the blood cells in the suspension above. As shown in **Fig. 6a–c**, clear detection of the targeted cells (both alive and dead) was possible. A dose-dependent response was again observed, with only 5–7% well-to-well variance. An EC_50_ value of 0.3 µg/mL was obtained (**Fig. 6d**), which was in line with that obtained in the CDC and ADCC assays. Thus, accurate data could be obtained without the use of PBMCs, which are typically only available in limited quantities. While CDC and ADCC assays will likely remain the principal means of evaluating new antibody candidates, this new whole blood assay can, in parallel, provide information on the performance of the agents in a more realistic sample matrix.

### Conclusion

There are number of significant advantages of this new cytotoxicity assay platform, including the use of smaller sample sizes, the immobilization of the cells through stronger linkages to minimize cell losses during the rinsing steps, the ability to obtain real-time data, and the ability to capture specific live cells that resist a given treatment method. Unlike most cytotoxicity assays, which indirectly relate cell death to the release of cytoplasmic contents into a bulk solution, this technique allows the researcher to obtain more accurate cytotoxicity data by directly reporting the number of living and dead cells. Such single-cell analysis capabilities [28], [29] were critically important to achieving the high precision observed in these assays, enabled the use of much smaller cell samples, and were required for the analysis of cytotoxicity in whole blood. This is particularly powerful when combined with the automated imaging and data analysis platforms that are available from a number of different vendors. The 96-well plate format allows replicate samples to be compared under uniform experimental conditions, as evidenced by the low variability between the wells in these experiments. Finally, the compatibility of the DNA-based adhesion strategy with both adherent and non-adherent cells allows these platforms to be extended for use in many different types of drug screens.

High-throughput cytotoxicity assays, such as these, could help to usher in a new era of personalized medicine. The small sample sizes and rapid analyses associated with this platform allow many more combinations of cell lines and drug candidates to be evaluated than were previously possible. This can facilitate the evaluation of combination therapies, in which two or more antibodies that target complementary receptor groups are used in concert. As each well requires 10,000 target cells or fewer, patient biopsies and primary leukemia cells isolated from blood samples can provide many more data points. This individualized screening process becomes particularly powerful when combined with the ability to evaluate the efficacy of a given agent in a patient’s own blood, likely allowing a more accurate prediction of how a therapeutic agent will perform once administered.

## Materials and Methods

### Preparation of Cell Microarrays

Cytolink cell microarray products were obtained from Adheren, Inc., in either 8-well chamber slide or 96-well microtiter plate formats. The cell microarrays were prepared following the protocol provided by the manufacturer. In brief, 60 µL of a 0.39 µM solution of DNA with a thiol at the 5′ end was reacted with 0.2 mg of succinimidyl-[(N- maleimidopropionamido)-hexaethyleneglycol] (NHS-PEO6-maleimide) (Pierce) in 40 µL of DMSO to obtain 1 mL of a 23.4 µM NHS-DNA solution in PBS (pH 7.2). The target cells, which had been rinsed twice with PBS before use, were then incubated with the NHS-DNA solution for 30 min at RT. Cell Tracker Blue or Green (Invitrogen, final concentration of 500 nM) was added along with the NHS-DNA solution if fluorescent labeling was needed. Following this, the cells were washed twice with PBS containing 1% FBS, and resuspended in a fresh portion of 1% FBS in PBS. In the case of 8-well chamber slides, a suspension of 1×10^7^ cells/mL was prepared, and 100 µL were added to each well. After 20 min of incubation at RT, the samples in either format were washed twice with PBS/1% FBS to remove any unbound cells. The cell microarrays were fully formed at this point, and could be observed and counted by microscopy or by using an automated imager.

For experiments that did not require the use of cell microarrays, 50 µL aliquots of the DNA-modified cells (5×10^5^ cells/mL) were added to each DNA complement-coated well. After 20 min of incubation at RT, all of the cells were bound to the surface. No further rinses were required before the addition of antibody solutions.

### Antibodies

The antibodies used in the CDC and ADCC assays were humanized anti-CD20 IgG_1_ (referred to as “α-CD20”) and ET901, which was raised against a proprietary non-mammalian target (used as a control, listed as “NC Ab”). Both were produced in CHO cells and obtained from Eureka Therapeutics as solutions in PBS and then diluted with PBS to generate a 2 mg/mL stock solution. Portions of this solution were further diluted as required before addition to the sample wells.

For experiments requiring fluorescent antibodies, the immunoglobulins were labeled with fluorescein isothiocyanate (FITC) and purified using gel filtration (NAP5 column, GE Healthcare) before use.

### Target Cell Preparation

The lymphoma cell line Jeko-1 was chosen for the ADCC and CDC experiments, and Jurkat leukemia T-cells (which are CD20 negative) were chosen as a control cell line. Whole blood cytotoxicity assays used Raji lymphoma cells. All cell lines were obtained from the ATCC and maintained in RPMI 1640 culture medium, supplemented with 10% heat-inactivated fetal bovine serum (FBS), 4 mM L-glutamine, and 500 IU/mL penicillin/streptomycin (Life technologies) at 37°C in a 5% CO2 incubator. Harvested cells were labeled with Cell Tracker Green or Cell Tracker Blue in accordance with the manufacturer’s instructions. The target cells were then immobilized on Cytolink cell microrarray chamber slides or in 96-well plates, as described above.

### CDC Assays

Jeko-1 lymphoma cells were immobilized on Cytolink cell microarrays in 8-well chamber slides. The assay buffer consisted of RPMI +0.1% BSA +20 mM HEPES. The antibody was dissolved in the assay buffer to form a series of stock solutions at twice the desired final concentrations (which resulted in 0.01 to 100 µg/mL concentrations after dilution in the sample wells). After the Jeko-1 cells had been bound by the microarray and the free cells had been removed by rinsing, 200 µL of the antibody solution was added to each well of the chamber slide. The resulting samples were incubated on ice for 60 min. After this time, 200 µL of prepared 30% (v/v) human serum solution in assay buffer was added to each chamber of the slide. Each sample was then incubated at 37°C with 5% CO_2_ for 4 h. Cell viability was analyzed using a propidium iodide (PI) solution (final concentration of 500 nM in each well), and imaged using a fluorescence microscope or an ImageXpress Micro (IXM) automated imager. The PI solution was added at the end of the experiments, or it was added with the serum if real-time images were to be collected.

### Antibody Resistance Assays

Jeko-1 lymphoma cells were immobilized on Cytolink cell microarrays in 8-well chamber slides. The assay buffer consisted of RPMI +0.1% BSA +20 mM HEPES. The FITC labeled α-CD20 antibody was dissolved in the assay buffer to form a stock solution at twice the desired final concentration (which resulted in 100 µg/mL after dilution in the sample wells). After the Jeko-1 cells had been bound by the microarray and the free cells had been removed by rinsing, 200 µL of the antibody solution was added to each well of the chamber slide. The resulting samples were incubated on ice for 60 min. After this time, 200 µL of prepared 30% (v/v) human serum solution in assay buffer was added to each chamber of the slide. Each sample was then incubated at 37°C with 5% CO2 for 4 h. Cell viability was analyzed using a propidium iodide (PI) solution (final concentration of 500 nM in each well), and imaged using a fluorescence microscope or an ImageXpress Micro (IXM) automated imager.

### Effector Cell Preparation

The effector cells that were used were fresh PBMCs obtained from AllCells, Inc. The effector cells were resuspended in RPMI 1640 culture medium, supplemented with 10% heat-inactivated fetal bovine serum (FBS), 4 mM L-glutamine, and 500 IU/mL penicillin/streptomycin. Cell viability was determined by 0.4% trypan blue exclusion before use.

### ADCC Assays

Jeko-1 lymphoma cells were immobilized on Adheren Cytolink cell microarrays in 8-well chamber slides. Before use, the Jeko-1 cells were stained fluorescent green using Cell Tracker Green. The assay buffer consisted of RPMI +10% FBS +1% penicillin/streptomycin. The antibody was dissolved in the assay buffer to form a series of solutions at twice the intended final concentrations (which resulted in 0.01 to 100 µg/mL concentrations after dilution in the sample wells). After the cells had bound the microarray surface and any free cells had been removed via rinsing, 200 uL of antibody solution was added to each of the wells. The number of Jeko-1 cells in each well was then counted using the IXM automated imager or a fluorescence microscope. The samples were next incubated at 37°C for 20 min. Fresh human PBMCs in assay buffer were prepared at a concentration of 1×10^6^ cells/mL. The desired volume of PBMCs was added to the wells to create a ratio of 25∶1 PBMCs to Jeko cells (E/T ratio = 25∶1). The chamber slide was then incubated at 37°C with 5% CO2 for 16 h. The samples were washed gently three times to remove most of the unbound effector cells, although this step was not necessary in all cases. Cell viability was analyzed using PI staining (500 nM final concentration), followed by cell counting with a microscopy imager (IXM) or a fluorescence microscope.

### Whole Blood Cytotoxicity Assays

Raji lymphoma cells were immobilized on Adheren Cytolink 96-well plates. In these experiments, the microarray cell patterns were not used. The target cells were stained fluorescent green before use with Cell Tracker Green. The assay buffer consisted of RPMI +10% FBS +1% penicillin/streptomycin (Life technologies). The antibody was dissolved in assay buffer to form a series of solutions at ten times the intended final concentrations (which resulted in 0.025 to 100 µg/mL concentrations after dilution in the sample wells). After the cells had bound the DNA-coated well plates (no rinsing was required), 20 µL of antibody solution was added to each of the sample wells. The plate was then incubated at 37°C for 20 min. A sample of heparinized fresh human whole blood was obtained from Allcells, Inc. To each sample well was added 180 uL of whole blood, and the resulting plate was then incubated at 37°C with 5% CO2 for 16 h. After this time, cell viability was analyzed directly (without rinsing away the effector cells) using a PI stain (500 nM final concentration). The cells were then imaged using a microscopy imager (IXM) or a fluorescence microscope.

## Supporting Information

Video S1 Time-lapsed images of the CDC process. Cell death was monitored for 4 hours using the dose of α-CD20 (10 µg/mL).
(WMV)
